# Understanding Changes in Adolescent Physical Activity Behaviors and Cognitions Prior to and During the COVID-19 Pandemic

**DOI:** 10.3389/fspor.2022.895097

**Published:** 2022-07-08

**Authors:** Dusan Kovacevic, Steven R. Bray, Denver M. Y. Brown, Matthew Y. W. Kwan

**Affiliations:** ^1^Department of Kinesiology, McMaster University, Hamilton, ON, Canada; ^2^Department of Psychology, University of Texas at San Antonio, San Antonio, TX, United States; ^3^Department of Child and Youth Studies, Brock University, St. Catharines, ON, Canada

**Keywords:** Physical activity cognitions, Physical activity, behavior change, youth, multi-process action control framework

## Abstract

Despite accumulating evidence that has found significant negative declines in physical activity (PA) as a result of the COVID-19 pandemic, little work has sought to understand how PA cognitions have changed during this period and in relation to behavior change during the pandemic. The purpose of the current study was to investigate the changes in adolescents' PA behaviors and cognitions associated with COVID-19 and prospective predictors of PA using the Multi-Process Action Control (M-PAC) framework. Adolescents were recruited from a large school board and a total of 588 participants (M_age_ = 15.87 ± 0.43 years, 60% female) completed data collection in both Fall 2019 and 2020—prior to and during the COVID-19 pandemic. Participants completed self-reported measures of moderate-to-vigorous PA (MVPA), participation in organized activities, and variables derived from the M-PAC framework. Mixed effects models were computed to examine longitudinal changes in MVPA and cognitions as well as whether cognitions prior to COVID-19 predict MVPA during COVID-19. A generalized estimating equations model was computed to examine longitudinal changes for participation in organized activities. Findings indicated that MVPA (B = −56.41, *p* < 0.01) and participation in organized activities (OR = 0.33, *p* < 0.01) significantly decreased during the COVID-19 pandemic. Correspondingly, there were significant decreases in intentions (B = −0.34), identity (B = −0.19), and habit (B = −0.20), though there were increases in behavioral regulation (B = 0.18). No significant changes were found in affective attitudes, instrumental attitudes, perceived opportunity, and perceived capability. Among the baseline M-PAC cognitions, habit (B = 46.28) was the lone significant predictor of MVPA during COVID-19. Overall results suggest that adolescents' PA behaviors and cognitions were negatively impacted by the COVID-19 pandemic, along with promising evidence that restrictions prompted adaptive utilization of behavioral regulation skills. Moreover, habit appears to play a salient role in predicting PA behaviors during uncertain times involving major life disruptions. These findings highlight the need to dedicate additional attention to PA promotion as COVID-19 moves toward an endemic phase, and that interventions targeting habit formation may be critical for helping adolescents better sustain healthy active lifestyles during major life changes.

## Introduction

Due to the alarming levels of viral transmission, severity, and inaction, the coronavirus disease 2019 (COVID-19) was declared a global pandemic in March 2020 (World Health Organization, 2020). In an effort to curb the spread of COVID-19, many jurisdictions introduced a number of public health measures in the forms of social distancing, self-isolation, and lockdown protocols. Preventive measures in the province of Ontario in Canada as an example, included at different times the closure of all non-essential workplaces, limits on community and social gatherings, requirements of physical distancing (staying at least 6 feet or 2 m apart from others), and limits and/or intermittent closures of recreational facilities (Government of Ontario, 2020). Although such measures have been necessary for controlling infection rates, these were also linked to significant changes in the daily lives of many (Constant et al., 2020; Ma et al., 2020).

One major consequence with the various restrictions has been the negative impact on physical activity (PA) opportunities (Stockwell et al., 2021). Indeed, this effect was illustrated among users of activity monitoring devices and apps such as Fitbit and Argus Azumio, which saw users accruing on average significantly fewer steps per day, particularly during the early part of the pandemic (Fitbit, 2020; Tison et al., 2020). For example, Tison et al. (2020) reported a 27.3% decrease in mean steps (1,432 steps) within 30 days of the pandemic declaration worldwide. Specific to the youth population, there has also been evidence that the pandemic has had a negative impact on their PA engagement. A study by Chaffee et al. (2021) found that among a sample of adolescents from Northern California, 54% reported being physically active 5 or more days per week prior to the COVID-19 pandemic compared to only 38% during the pandemic. Similarly, in Canada, Moore et al. (2020) found that youth reported declines in PA during the COVID-19 outbreak, with the greatest declines specific to outdoor PA and organized sport. More recently, a systematic review by Stockwell et al. (2021) found six studies focusing on healthy children and adolescent populations—all reporting decreases in PA participation regardless of the measurement tool used or how PA was operationalized. This downward trend in PA is particularly alarming given that only 30% of Canadian youth (12- to 17-year-olds) were achieving the recommended amount of PA [at least 60 min of daily moderate-to-vigorous PA (MVPA)] prior to the pandemic (ParticipACTION, 2020). These further reductions in PA behaviors will likely deny many additional youth the well-established health benefits associated with regular PA engagement (Janssen and LeBlanc, 2010; Poitras et al., 2016; ParticipACTION, 2020).

Given the ongoing uncertainties associated with COVID-19 restrictions, there is a need for initiatives that promote effective adaptation to changing social and environmental factors to help sustain PA behaviors. Despite accumulating evidence reinforcing the negative declines in PA corresponding with the pandemic (Stockwell et al., 2021; Wunsch et al., 2022), little work has sought to understand how PA cognitions have changed during this same period and their relationship to PA behaviors through COVID-19. Longitudinal investigations of the changes in PA-related cognitions and PA behaviors are needed to help inform strategies for interventions targeting adolescent PA behaviors. Implications of this may not only be relevant for the ongoing changes with COVID-19, but potentially for other abrupt life changes such as for adolescents' transition toward emerging adulthood.

The multi-process action control (M-PAC) framework (Rhodes, 2017) was recently developed to represent a range of capability, opportunity, and motivational constructs (COM-B; Michie et al., 2011) that are commonly represented in theories of behavior and behavior change. The M-PAC framework also augments well-established antecedents of PA behavior with constructs to help bridge the intention-behavior gap. According to the M-PAC framework, PA is influenced by three core processes: reflective processes to represent the consciously deliberated expected consequences of performing PA and the act of behavioral performance; regulatory processes to represent the behaviors or cognitions that people enact to translate their initial intentions into PA behavior; and reflexive processes to denote the impulsive and automatic processes involved in behavioral action. Reflective processes include affective attitudes (expected feelings during PA), instrumental attitudes (expected utility of engaging in PA), perceived opportunity (time and access to engage in PA), and perceived capability (perceptions of ability to engage in PA). These constructs are considered antecedents of intention formation. Regulatory processes include goal setting, self-monitoring, action planning, and coping planning, which are collectively categorized as behavioral regulation. There are two primary reflexive processes: habit (routine behavioral action executed from cues) and identity (association with a particular role through self-categorization).

The M-PAC framework has been employed to describe, explain, and predict PA as well as develop interventions to promote PA (Quinlan et al., 2015; Rhodes et al., 2016a, 2019, 2020a; Vallerand et al., 2016, 2017; Kaushal et al., 2017; Liu et al., 2019; Brown et al., 2020; Kwan et al., 2020, 2021; Grant et al., 2021). Given the breadth of reflective, regulatory, and reflexive constructs represented in the model, M-PAC offers a comprehensive framework to examine how adolescents' PA cognitions may have changed as a result of the COVID-19 pandemic, and whether their cognitions prior to the pandemic may predict PA behavior during the pandemic. By employing the M-PAC framework, we may provide novel insights into the impact of the COVID-19 restrictions on attitudes toward PA, perceived behavioral control over being active, behavioral regulation skills, as well as more automatic processes such as identity and habit. The comprehensiveness of the M-PAC framework is particularly useful for the context of this study given that many of the constructs may be sensitive to environmental perturbations such as the pandemic restrictions.

The purpose of the present study was two-fold: (1) to utilize the M-PAC framework to investigate changes in adolescents' MVPA behaviors and PA-related cognitions during the second wave of the COVID-19 pandemic in Ontario, Canada and (2) to investigate whether PA-related cognitions prior to COVID-19 predicted MVPA during the pandemic. Based on prior research showing PA declined among children and youth during the pandemic (Moore et al., 2020; Chaffee et al., 2021; Stockwell et al., 2021), it was hypothesized that adolescents' MVPA would be lower during the midst of pandemic compared to pre-pandemic levels. Additionally, given the cancellation of many organized activities during the pandemic (Government of Ontario, 2020) and declines in outdoor PA and sport (Moore et al., 2020), it was hypothesized that there would be a significant decrease in reported organized activities during the pandemic. Resultant of the many restrictions during the pandemic, it was also hypothesized that we would see significant changes in many of the M-PAC-based reflective, regulatory, and reflexive processes. Given that the factors of behavioral regulation, identity, and habit were found to be salient predictors of PA among adolescents prior to the onset of the pandemic (Kwan et al., 2021), it was also hypothesized that these factors would be significant predictors of MVPA during the pandemic.

## Materials and Methods

### Study Sample and Data Collection

Data for the present study come from the first two annual data collection cycles of the prospective cohort study: the Application of integrateD Approaches to understanding Physical activity during the Transition to emerging adulthood (ADAPT study). Baseline data were collected from grade 11 students across a large school board (consisting of seven secondary schools) in Ontario, Canada. Participation was voluntary and informed consent was obtained from both students and parents prior to data collection. Students willing to participate completed a 20-min online questionnaire that included questions related to lifestyle behaviors, PA cognitions, and wellbeing during class time in Fall 2019—prior to the pandemic. A follow-up of these students occurred ~12 months later in Fall 2020—during which pandemic-related restrictions were in place. Participants were compensated with a $10 gift card for their participation at each time point. All protocols for the ADAPT study were approved by the institutional research ethics board and district school board. Further details regarding recruitment strategy and research design are reported elsewhere (Kwan et al., 2020).

From the 2,412 grade 11 students enrolled in the seven secondary schools within the school board, 1,585 agreed and provided consent to participate (66% response rate). Of these students, 146 respondents (9%) completed <5% of the survey and their participation was considered withdrawn, while 186 respondents (12%) did not have the required parental consent for their participation. Further, 15 respondents (1%) were missing data for all variables of interest used in our analyses. Among the baseline sample of 1,238 participants, 588 (*M*_*age*_ = 15.87 ± 0.43 years at baseline) also completed the follow-up questionnaire (retention rate = 47.5%) and this subsample was used for the present study. The overall sample included a slightly greater proportion of females (*n* = 350; 60%) than males (*n* = 236; 40%) and slightly more than half the sample identified as White/Caucasian (54%). Complete details of demographic information are shown in Table 1.

**Table 1:** Baseline demographic characteristics.

**Characteristics**	**Total sample (*n* = 1,238)**	**Only baseline (*n* = 650)**	**Both assessments (*n* = 588)**	***p*-value**
Mean age in years (SD)	15.91 (0.50)	15.94 (0.55)	15.87 (0.43)	
Gender				<0.001
Male	569 (46%)	333 (51%)	236 (40%)	
Female	651 (53%)	301 (46%)	350 (60%)	
Prefer not to answer/missing	18 (1%)	16 (2%)	2 (0.3%)	
Ethnicity				0.25
White/Caucasian	642 (52%)	327 (50%)	315 (54%)	
Non-white/non-Caucasian	596 (48%)	323 (50%)	273 (46%)	

*Differences in baseline characteristics between those that participated in only the baseline assessment or both assessments were examined by chi-square tests. SD, standard deviation*.

### Measures

#### Demographics

Participants completed demographic questions assessing age, gender, and ethnicity. For analyses purposes, gender (male or female) and ethnicity (White/Caucasian or non-White/non-Caucasian) were dummy coded, with males and White/Caucasian as the reference group.

### Physical Activity Behaviors

#### Moderate-to-Vigorous Physical Activity

MVPA was measured using the International Physical Activity Questionnaire-Short Form (IPAQ-SF; Booth, 2000; Craig et al., 2003). Participants responded to 2 items assessing how many days they did (1) moderate-intensity PA and (2) vigorous-intensity PA over the past 7 days. If they indicated that they participated in an activity for at least 1 day, they then reported how much time (in hours and/or minutes) they usually spent on one of those days participating in that activity. Weekly MVPA was calculated by multiplying frequency by duration for both moderate PA and vigorous PA, and then summing the products. As per the scoring rules for IPAQ, daily MVPA times were capped to 180 min for any participants who exceeded 3 h or 180 min of MVPA per day. The IPAQ-SF has shown acceptable measurement properties when administered among adolescents (Guedes et al., 2005).

#### Participation in Organized Activities

Participation in organized activities (yes/no) was assessed by asking participants if they are presently enrolled in organized activities (i.e., sports team, intramurals, scheduled physical activity classes, etc.).

### Physical Activity Cognitions

#### Behavioral Intention

Behavioral intention for engaging in MVPA was assessed using a single item measure (Courneya, 1994). Participants were asked to rate the extent to which the following statement is likely: “I intend to do at least 150 min per week of moderate-to-vigorous physical activity.” Responses were rated on a 7-point scale ranging from 1 (extremely unlikely) to 7 (extremely likely).

#### Affective Attitudes

Affective attitudes were assessed using 3 items presented on a 7-point bipolar scale (Rhodes and Courneya, 2003). Items were prefaced with the stem “Over the next 4 weeks, being physically active, for me, will be….” The items used were: unpleasant-pleasant, boring-fun, and unenjoyable-enjoyable. Items were averaged to provide a score ranging from 1 to 7 (Cronbach's α's > 0.88–0.89).

#### Instrumental Attitudes

Instrumental attitudes were assessed using 3 items presented on a 7-point bipolar scale (Rhodes and Courneya, 2003). Items were prefaced with the stem “Over the next 4 weeks, being physically active, for me, will be….” The items used were: harmful-beneficial, bad-good, and useless-useful. Items were averaged to provide a score ranging from 1 to 7 (Cronbach's α's > 0.91).

#### Perceived Opportunity

Perceived opportunity for engaging in regular PA was measured by asking participants to what extent they agree with 3 items (Rhodes et al., 2006). Items were rated on a 7-point scale ranging from 1 (strongly disagree) to 7 (strongly agree). The items were: (1) “If I really wanted to do regular physical activity over the next 4 weeks, I would have a chance to do so,” (2) “I lack the opportunity to do regular physical activity over the next 4 weeks, even if I were really motivated to do so,” and (3) “There are places where I can do physical activity around me if I had to.” Items were averaged to provide a score ranging from 1 to 7 (Cronbach's α's > 0.63).

#### Perceived Capability

Perceived capability for engaging in regular PA was measured by asking participants to what extent they agree with 3 items (Rhodes et al., 2006). Items were rated on a 7-point scale ranging from 1 (strongly disagree) to 7 (strongly agree). The items included: (1) “I possess the skills to do regular physical activity over the next 4 weeks if I wanted to,” (2) “I have the physical ability to do regular physical activity over the next 4 weeks if I wanted to,” and (3) “I am confident that I am capable of engaging in regular physical activity if I had to.” Items were averaged to provide a score ranging from 1 to 7 (Cronbach's α's > 0.90–0.91).

#### Behavioral Regulation

Behavioral regulation was assessed using the Behavioral Regulation for Physical Activity Scale (Sniehotta et al., 2005), consisting of 6 items rated on a 5-point scale ranging from 1 (strongly disagree) to 7 (strongly agree). The items were: (1) “I kept track of my physical activity in a diary or log over the last month,” (2) “I set short-term (daily or weekly) goals for leisure-time physical activity last month,” (3) “I made regular plans concerning ‘when', ‘where', ‘how', and ‘what' kind of physical activity I did last month,” (4) “I made plans regarding what to do if something interfered with my engaging in physical activity,” (5) “I reserved time in my daily schedule for regular leisure-time physical activity,” and (6) “If I did not reach a physical activity goal, I analyzed what went wrong.” Items were averaged to provide a score ranging from 1 to 7 (Cronbach's α's > 0.86).

#### Physical Activity Identity

Identity was assessed using an abbreviated version of the Exercise Identity Scale (Anderson and Cychosz, 1994), asking participants how their PA is related to their concept of self. This scale consisted of 5 items that were rated on a 7-point scale ranging from 1 (strongly disagree) to 7 (strongly agree). Example items include “I consider myself as someone who is physically active” and “Others see me as someone who does physical activity regularly.” Items were averaged to provide a score ranging from 1 to 7 (Cronbach's α's = 0.91–0.92).

#### Habit of Physical Activity

Habit for engaging in PA was assessed using the Self-Report Habit Index (Gardner et al., 2012), which asks participants to what extent physical activity is a habit for them. This index consists of 4 items that are rated on a 7-point scale ranging from 1 (strongly disagree) to 7 (strongly agree). Items were prefaced with the stem “Regular physical activity is something….” Example items include “… I do automatically” and “… I do without having to consciously remember.” Items were averaged to provide a score ranging from 1 to 7 (Cronbach's α's > 0.90–0.91).

#### Data Analysis

For the longitudinal analysis of participation in organized activities, a generalized estimating equations model was used. Generalized estimating equations is a technique used for modeling repeated binary observations within individuals, and our model was adjusted for gender and ethnicity. Separate mixed effects models with maximum likelihood were computed to measure change within individuals over time in MVPA and across each PA cognition, accounting for correlation of measures within participants (repeated measures) and within schools. All models included random intercepts at the participant and school levels, as well as a random slope for time. Each model was adjusted for gender and ethnicity. Mixed effects models with PA cognitions as the outcome variable were also adjusted for baseline MVPA. To examine whether M-PAC cognitions at baseline predicted MVPA at follow-up, a 2-step mixed effects model was computed with the potential covariates: gender, ethnicity, and baseline MVPA included on Step 1, and baseline M-PAC cognitions entered on Step 2. Given that separate mixed effects models were computed for each of the eight PA cognitions identified in the M-PAC framework, a Bonferroni correction was applied to limit the Type I error rate. Therefore, statistical significance was set at *p* <0.006 (0.05/8) for the analyses involving PA cognitions. Statistical significance was set at *p* < 0.05 for all other analyses. All analyses were performed using IBM SPSS version 26.

## Results

The descriptive statistics for PA behaviors and PA cognitions at the baseline and follow-up assessments are presented in Table 2.

**Table 2:** Descriptive statistics for PA behaviors and PA cognitions at baseline and follow-up.

**Variable**	**Total sample**		**Completed both assessments**			
	**Baseline**		**Baseline**		**Follow-up**	
	***n***	**M (SD) or *n* (%)**	***n***	**M (SD) or *n* (%)**	***n***	**M (SD) or *n* (%)**
PA behaviors						
MVPA (mins/week)	1233	579.23 (413.90)	585	536.81 (391.14)	584	498.18 (448.27)
Participation in organized activities	1223	714 (58%)	583	346 (59%)	584	191 (33%)
Reflective processes						
Behavioral intention	1177	5.16 (1.83)	559	5.18 (1.78)	541	4.75 (1.89)
Affective attitudes	1234	5.20 (1.36)	587	5.16 (1.36)	585	5.02 (1.41)
Instrumental attitudes	1234	6.01 (1.13)	587	6.05 (1.11)	585	5.96 (1.19)
Perceived opportunity	1198	5.46 (1.25)	587	5.48 (1.27)	587	5.28 (1.32)
Perceived capability	1232	5.82 (1.30)	587	5.83 (1.29)	587	5.73 (1.37)
Regulatory processes						
Behavioral regulation	1216	2.61 (0.99)	588	2.62 (0.99)	588	2.76 (1.07)
Reflexive processes						
Identity	1236	4.62 (1.70)	588	4.59 (1.68)	587	4.37 (1.71)
Habit	1236	4.43 (1.61)	588	4.38 (1.59)	587	4.15 (1.64)

*M, mean; SD, standard deviation*.

### Changes in Physical Activity Behaviors

Results from the generalized estimating equations model showed a significant negative effect of time (OR = 0.33, 95% CI [0.28–0.40], *p* < 0.001), indicating that participants were 67% less likely to participate in organized activities during the pandemic compared to before the pandemic. Descriptively, among participants who completed the survey at both time points, 59% of participants (*n* = 346) reported engaging in organized activities prior to the pandemic, while only 33% of participants (*n* = 191) indicated that they engaged in organized activities during the pandemic as seen in Table 2. Results from the mixed effects model for MVPA also showed a significant effect for time (Estimate = −56.41, SE = 19.54, *p* = 0.004), indicating a reduction of 56.41 min of MVPA during the pandemic compared to before the pandemic.

### Changes in Physical Activity Cognitions

Results from our mixed effects models examining changes in PA cognitions found several significant changes over time. Among the reflective processes, PA intention (B = −0.34, SE = 0.09, *p* < 0.001) significantly decreased over time. Affective attitudes (B = −0.11, SE = 0.06, *p* = 0.06), instrumental attitudes (B = −0.04, SE = 0.05, *p* = 0.46), perceived opportunity (B = −0.16, SE = 0.06, *p* = 0.009), and perceived capability (B = −0.04, SE = 0.06, *p* = 0.50) did not significantly change. For regulatory processes, behavioral regulation (B = 0.18, SE = 0.04, *p* <0.001) was found to have significantly increased over time. Similarly, both reflexive process measures of identity (B = −0.19, SE = 0.06, *p* = 0.002) and habit (B = −0.20, SE = 0.06, *p* = 0.002) were significant, decreasing between baseline and follow-up. These findings indicate that adolescents reported lower PA intentions, identity, and habit as well as greater behavioral regulation during the pandemic compared to before the pandemic.

### Predictors of Moderate-to-Vigorous Physical Activity During COVID-19

Complete results of the mixed effects model predicting MVPA during COVID-19 are presented in Table 3. Among all the baseline M-PAC predictors entered in the model after controlling for covariates, only habit was found to be a significant predictor (B = 46.28, SE = 18.45, *p* = 0.01). This finding indicates that adolescents who reported stronger PA habits prior to the COVID-19 pandemic engaged in greater amounts of MVPA during the pandemic. No reflective or regulatory measures were found to be significant predictors.

**Table 3:** Results from mixed effects model predicting MVPA at follow-up from predictors at baseline.

	**Step 1**	**Step 2**
Intercept	410.01 (44.29)	230.35 (121.63)
Covariates		
Gender	−155.71^**^ (37.12)	−133.60^**^ (37.26)
Ethnicity	−2.59 (35.65)	4.05 (35.43)
Baseline MVPA	0.33^**^ (0.05)	0.23^**^ (0.05)
Reflective processes		
Behavioral intention		8.03 (14.69)
Affective attitudes		32.21 (20.80)
Instrumental attitudes		−14.31 (22.09)
Perceived opportunity		−5.91 (18.23)
Perceived capability		−11.37 (20.77)
Regulatory processes		
Behavioral regulation		9.25 (21.77)
Reflexive processes		
Identity		−6.51 (19.79)
Habit		46.28^*^ (18.45)
R^2^	0.13^**^	0.17^**^
Change in R^2^		0.04^**^

*Standard error in parentheses*.

* *p < 0.05*;

** *p < 0.01*.

## Discussion

The present study assessed changes in Canadian adolescents' PA behaviors from pre- to mid-pandemic (Fall 2019–Fall 2020) and represents the first to investigate changes in PA-related cognitions during this timeframe. Our findings showed that adolescents spent less time engaging in MVPA, while also being less likely to engage in organized activities during the COVID-19 pandemic compared to pre-pandemic levels. In addition, we found that there were significant declines in intentions, identity, and habit as well as increases in behavioral regulation. Importantly, findings indicate that pre-pandemic PA habits were significant in predicting MVPA during the pandemic.

Overall, our results suggest that pandemic-related restrictions negatively impacted adolescents' PA behaviors during the first year of the COVID-19 pandemic, which is largely consistent with previous studies (Moore et al., 2020; Chaffee et al., 2021; Stockwell et al., 2021). This observed reduction in MVPA along with participation in organized activities is likely a result of many cancellations in youth sport programs, restrictions of school-supported extracurricular activities, changing rules or access to community facilities, and potentially decreases in active transportation with many students having to adjust to a blend of virtual learning at home and in-person learning at school. PA is an important component to adolescent health as greater PA levels in youth are associated with better physical health, mental health, cognitive functioning, and academic achievement (Warburton et al., 2006; Donnelly et al., 2016; Biddle et al., 2019). Importantly, establishing healthy PA behaviors during the adolescent period has significant implications for future health risks, as those that become inactive as they enter adulthood are at greater risk of cardiovascular risk factors and obesity (Tammelin et al., 2004). Consequently, declining adolescent PA behaviors due to the COVID-19 pandemic may have significant future health implications given that PA tracks from adolescence into adulthood (Kwon et al., 2015). It should be noted, however, that despite overall mean decreases in MVPA, a sizable proportion of our adolescent sample (40%) did report increases in their MVPA through the COVID-19 pandemic. Further research investigating the factors related to different behavioral trajectories appear warranted.

Despite studies consistently demonstrating reductions in PA behaviors since the onset of the COVID-19 pandemic (Stockwell et al., 2021; Wunsch et al., 2022), minimal work has investigated psychological factors that may help to explain these changes. To the best of our knowledge, the present study represents the first to examine changes in adolescent PA-related cognitions associated with the onset of the COVID-19 pandemic. Among the reflective processes investigated, only intentions significantly decreased during the pandemic. While not always a strong predictor of actual behaviors, intentions are still weak-to-moderately related to PA (Rhodes, 2017). Perhaps more importantly, intentions are considered a necessary condition for most people to engage in PA behaviors (Rhodes and de Bruijn, 2013).

Attitudes and perceived capabilities are posited to play a role in intention formation and should remain stable even in the face of major changes; however, it was interesting that perceived opportunities also did not change significantly from pre- to being in the midst of the pandemic. This finding is in contrast to a study by Spence et al. (2021), which found that British adults who perceived to have greater physical opportunities and motivation to be active were more likely to maintain or increase their PA during the COVID-19 lockdown. One potential explanation for the difference in findings is that data collection in Spence et al. occurred in the immediate months once COVID-19 was declared a pandemic, whereas data for this study was collected several months later into the Fall. Respondents' perception of opportunities to be active may have been different at these time points. Additionally, opportunities to be active provided by schools and neighborhoods may be different in the UK compared to Ontario. In sum, given that attitudes and perceived behavioral control did not appear to be affected in the early stages of the pandemic, findings suggest other factors such as subjective norms, which was not accounted for in this study, likely impacted the intention formation process.

Another noteworthy finding was the increase in behavioral regulation during the COVID-19 pandemic. These results suggest that the restrictions prompted adaptive utilization of behavioral regulation skills, such as action and coping planning to schedule and deal with potential or anticipated problems. Given that many of the restrictions imposed likely caused disruptions to typical behaviors, adolescents had to strategically formulate and implement new plans to be physically active. These findings suggest that in addition to efforts to help increase PA intentions that may have declined during the pandemic, more emphasis should be placed on helping adolescents regulate their behaviors through self-monitoring, goal setting, planning, and coping with major changes so that they can be more resilient and bridge the intention-action gap, regardless of their dynamic circumstances.

Invariably, major changes often accompany adolescents' transition out of high school, and along with intentions and perceived opportunity, interventions targeting identity and habit formation and re-formation will be critical. What was apparent was that the COVID-19 pandemic drastically changed adolescents' regular lifestyle routines, likely impacting their PA habits and identities related to PA at the same time. For example, many adolescents who self-categorize as a physically active person may not have been able to do the same activities during the pandemic and found themselves less likely to identify as that same regularly active person. Similarly, if an individual's contextual cue that prompts PA is no longer present, such as the cancellation of their soccer league, their habits are disrupted and PA habits diminished. This study was the first to investigate identity; however, declines in PA habits during the COVID-19 lockdown were also reported in a sample of adults from France and Switzerland (Maltagliati et al., 2021). Reflexive processes have attracted increased attention over the past decade (Rhodes et al., 2016b; Gardner et al., 2021), yet there is still considerable work to be done to improve our understanding of how to maintain or re-form habits and identities that will enable adolescents to stay active during turbulent life events and times of stress.

Finally, our study examined how PA cognitions prior to the pandemic predicted PA behaviors during COVID-19. Results showed that habit was the lone significant predictor after controlling for several covariates, including pre-pandemic MVPA. Given that previous MVPA is a powerful predictor of future MVPA, and often attenuates the effects of psychosocial and personal predictors (Hagger et al., 2002), the additional variance explained by habit illustrates its importance in predicting future MVPA. Simply stated, adolescents with stronger PA habits prior to the pandemic engaged in more MVPA during the pandemic. This finding is largely consistent with previous studies that have reported habit to be an important predictor of changes in MVPA as a result of early pandemic restrictions (Rhodes et al., 2020b), and a salient predictor of adolescent PA (Kwan et al., 2021). While this recent study by Kwan et al. (2021) also found coping planning and identity to be significantly associated with MVPA among adolescents prior to the pandemic, these variables were not found to predict MVPA during mid-pandemic. Overall, these factors may not be as important as habit in protecting against PA declines during environmental perturbations such as pandemic restrictions. A greater understanding of habit formation and disruption will provide critical insight for the development of future interventions aiming to promote PA during adolescence (Gardner et al., 2021).

It is also important to note that the variance explained in the model was quite low. This may be due to the longitudinal design, where MVPA during COVID-19 was predicted by baseline M-PAC cognitions from 1 year prior. Availability of home PA equipment, dog ownership, personality traits, and COVID-19 related constructs such as social distancing behavior have been shown to correlate with MVPA during the pandemic and may account for more variance (Rhodes et al., 2020b). Another factor that might account for more variance in MVPA during COVID-19 is stress, which can impede efforts to engage in PA or may elicit increases in PA behaviors (Stults-Kolehmainen and Sinha, 2014).

Findings from this study have several implications for PA promotion. Notably, stronger intervention efforts are needed, particularly as adolescents transition into emerging adulthood throughout the next few years amidst the pandemic. Even prior to the pandemic, there has been an emergence of evidence suggesting that the transition from adolescence into emerging adulthood is a period of great risk of PA declines (Kwan et al., 2012; Corder et al., 2019; Gropper et al., 2020). Greater efforts must be taken to re-engage individuals in emerging adulthood that previously reported participation in organized activity during adolescence. These efforts may include health promotion messaging from public health officials, intentionally targeting the return to organized play for older youth, and better promotion of opportunities to engage in PA by post-secondary institutions for the many emerging adults that transition to higher education (Faulkner et al., 2020).

Although this study presents novel findings, there are several limitations that should be acknowledged. The first limitation is a temporal consideration, in that we were not able to capture the initial changes in PA behaviors and cognitions during the onset of the COVID-19 pandemic. By the time students were administered the questionnaire in Fall 2020, they may have adopted strategies to better cope with the unique challenges presented over the first 6 months of the pandemic. Second, PA behaviors were measured using self-report assessments, and are therefore susceptible to over-reporting due to social desirability biases and recall errors (Prince et al., 2008). However, the use of the IPAQ-SF as the self-report measure for MVPA is a relative strength considering it is one of the most commonly used self-report tools for PA in the literature, allowing for comparisons across studies. Third, while our study began with a large sample of adolescents across a large school board, the attrition at follow-up was larger than anticipated, particularly among male participants. The pandemic itself limited our abilities to follow-up with participants at school, which may have contributed to the overall low retention rate and may have disproportionately affected the retention of male participants. Thus, there was likely a response bias that could limit the generalizability of the study. Finally, given that this study did take place in a single geographic area in Ontario, these findings may not be applicable to other jurisdictions across other provinces or countries that may have had different COVID-19 restrictions.

In conclusion, this study provides some important insights into the changes in adolescents' PA behaviors and cognitions associated with COVID-19 as well as prospective predictors of MVPA during the pandemic. The findings from this study reinforce the need for public health strategies to help reverse the declines in PA behaviors and to make more concerted efforts to target PA among adolescents as they transition into emerging adulthood amidst the pandemic. Changes in several PA cognitions accompanied pandemic related declines in PA behaviors. School-based interventions should focus on helping adolescents maintain greater PA intentions and alongside strategies to reform strong PA habits and maintain identities. While the focus of this work was to understand changes that occurred with the COVID-19 pandemic, implications of this work extend to the major life changes that occur as adolescents transition beyond adolescence and into emerging adulthood.

## Data Availability Statement

The raw data supporting the conclusions of this article will be made available by the authors, without undue reservation.

## Ethics Statement

This study involved human participants and was reviewed and approved by the McMaster Research Ethics Board and the Hamilton-Wentworth Catholic District School Board Ethics Committee. Written informed consent to participate in this study was provided by study participants. Written informed consent to participate in this study was provided by the participants' legal guardian/next of kin.

## Author Contributions

DK: conceptualization, methodology, formal analysis, and writing—original draft. SB: methodology, writing—review and editing, and supervision. DB: methodology and writing—review and editing. MK: conceptualization, data curation, methodology, writing—review and editing, supervision, and funding acquisition. All authors contributed to the article and approved the submitted version.

## Funding

The ADAPT study was funded by an Insight Grant (Grant #435- 2018-0896) from the Social Sciences and Humanities Research Council of Canada.

## Conflict of Interest

The authors declare that the research was conducted in the absence of any commercial or financial relationships that could be construed as a potential conflict of interest.

## Publisher's Note

All claims expressed in this article are solely those of the authors and do not necessarily represent those of their affiliated organizations, or those of the publisher, the editors and the reviewers. Any product that may be evaluated in this article, or claim that may be made by its manufacturer, is not guaranteed or endorsed by the publisher.
